# Oral cavity metastasis of renal cell carcinoma: A case report

**DOI:** 10.1186/1752-1947-2-313

**Published:** 2008-09-29

**Authors:** Thomas Anthony Will, Neena Agarwal, Guy Joseph Petruzzelli

**Affiliations:** 1 Department of Urology, Loyola University Medical Center, 2160 S. First Ave., Bldg 54 – 2nd Floor, Maywood, IL 60153, USA; 2 Department of Otolaryngology, Loyola University Medical Center, 2160 S. First Ave., Maguire Bldg, Maywood, IL 60153, USA; 3 Rush University Medical Center, 1725 West Harrison Street, Professional Building 1, Suite 218, Chicago, IL 60612, USA

## Abstract

**Introduction:**

Despite being reported rarely, renal cell carcinoma is the third most frequent neoplasm to metastasize to the head and neck region preceded only by breast and lung cancer. Little information exists regarding the presentation and work-up of metastatic renal cell carcinoma in the oral cavity.

**Case presentation:**

We report the case of a 63-year-old Caucasian man presenting with an oral cavity lesion that was painful and that had grown substantially over several months. Biopsy resulted in persistent bleeding requiring cautery and manual pressure. Immunoperoxidase testing was necessary to make the diagnosis of metastatic renal cell carcinoma and rule out other clear cell carcinomas of salivary gland origin.

**Conclusion:**

Metastatic renal cell carcinoma is part of the differential diagnosis for patients presenting with a new head or neck lesion in the setting of a history of kidney cancer. The physician needs to be prepared for the increased risk of bleeding and understand the importance of immunohistochemical staining to differentiate between metastatic renal cell carcinoma and malignancies of salivary origin. Unfortunately, the prognosis is invariably poor in these patients.

## Introduction

Metastatic lesions of the oral cavity are extremely rare, accounting for approximately 1% of all malignant oral tumors. Renal cell carcinoma (RCC) is the third most frequent neoplasm to metastasize to the head and neck region preceded only by breast and lung cancer. It accounts for nearly 3% of all adult malignancies and is the most lethal urologic cancer. Approximately one-third of patients present with metastatic disease and 40% to 50% will develop distant metastases (asynchronous metastatic disease) after the initial diagnosis. The expected 5- and 10-year survival rates for these patients are 5–30% and 0–5%, respectively. The most common sites of metastasis include the lungs, regional lymph nodes, bone, liver, adrenal glands, contralateral kidney and brain [1].

Despite being reported infrequently, head and neck region metastases may be linked to RCC in up to 8–15% of cases [2]. The nose and paranasal sinuses are most commonly affected followed by the oral cavity. Within the oral cavity, the tongue is a frequent target for RCC metastasis while isolated spread to the floor of mouth is rarely reported. Lesions in the tongue or floor of mouth area can cause severe pain, bleeding, difficulty with eating and even complete oral obstruction. Unfortunately, oral cavity metastasis from RCC is usually a manifestation of widespread disease. The following is a case study of a patient with oral cavity metastasis of renal adenocarcinoma.

## Case presentation

A 63-year-old Caucasian man presented to his primary care physician with a 6-month history of intermittent right anterior neck and intraoral pain. The patient noted a tongue mass, which had grown substantially over the last several months. The mass made eating difficult at times and resulted in one episode of mild oral bleeding that resolved spontaneously. He was referred to our institution's department of otolaryngology/head and neck surgery for further evaluation.

The patient's past medical history is significant for RCC of the right kidney diagnosed 4 years prior and treated with right radical nephrectomy. An appropriate work-up at that time included a CT scan of the chest, abdomen, and pelvis and liver functions tests, all of which were negative for metastatic disease. He did not follow-up with his urologist as recommended after the surgery.

The physical exam revealed an erythematous, indurated 3 cm mass in the right anterior floor of mouth region that was tender to palpation. It was not fixed to the mandible and appeared vascular. The neck exam was positive for a 3 cm firm mass in the right thyroid lobe with no pathologic lymphadenopathy otherwise.

**Figure 1:**
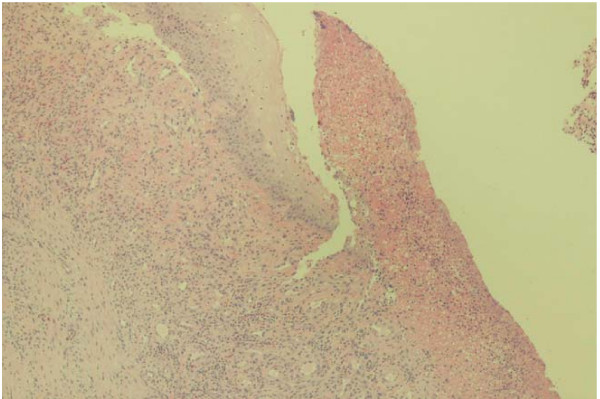
Renal cell carcinoma; ulceration of mucosal epithelium noted secondary to tumor cell infiltration.

**Figure 2:**
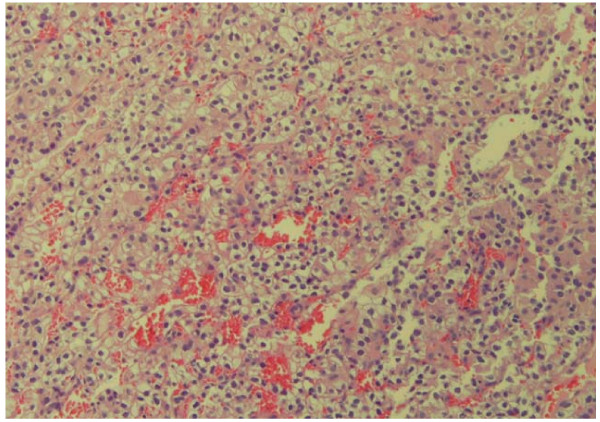
Histologic features of renal cell carcinoma; epithelial cellular network shown with clear cytoplasm and hyperchromatic nuclei surrounded in a rich vascular network.

**Figure 3:**
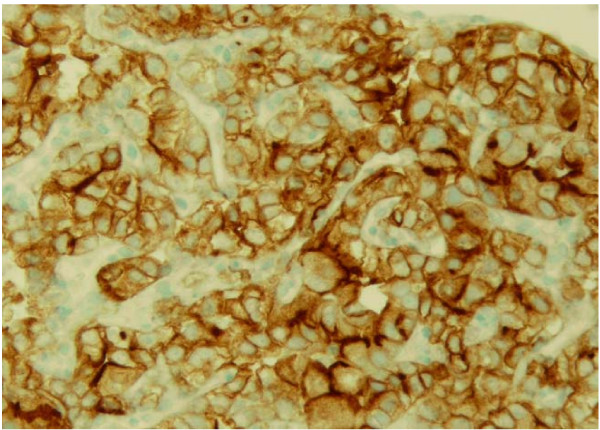
Staining for clear cell carcinoma; carcinomatous cells are positive for vimentin by immunohistochemical staining.

Biopsy of his anterior floor of mouth lesion was notable for persistent bleeding and revealed clear cell carcinoma, consistent with the patient's previous history of renal cell cancer (Figure 1). Histologic evaluation revealed the presence of a solid nest of epithelial cells with clear cytoplasm and small, round hyperchromatic nuclei (Figure 2). A rich vascular network was also noted. Immunoperoxidase testing was positive for CD10 and vimentin and negative for gross cystic disease fluid protein (GCDFP), S-100, HMB-45, muscle-specific antigen, and desmin, supporting the diagnosis of metastatic RCC (Figure 3).

Original surgical, pathology and postoperative records were eventually obtained revealing the discovery of suspicious lymph nodes near the renal hilum during his original nephrectomy. The resected lymph nodes were found to harbor metastatic carcinoma and the patient was referred to a medical oncologist at that time to discuss additional therapeutic options. Unfortunately, he did not seek further care for his metastatic RCC. After receiving the news of his biopsy results in our clinic, the patient decided to return to his original institution for further care and died of metastatic disease within several months.

## Discussion

Metastatic tumors to the oral cavity are extremely rare. Therefore, existing literature is based largely on sporadic case reports. Possible routes of metastasis to the oral cavity include arterial, venous and lymphatic circulations. In the case of head and neck metastasis without lung involvement, several theories exist to address a route of dissemination that avoids pulmonary vascular filtration. These include spread via Batson's venous plexus or through the thoracic lymphatic duct. Batson's paraspinal plexus is a valveless, venous system extending from the skull to the sacrum allowing tumor emboli to bypass the pulmonary venous system with minimal resistance resulting in metastasis to the head and neck region in the absence of obvious lung lesions [3].

Reports of RCC to the head and neck region involve the nose, tongue, paranasal sinuses, larynx, mandible, temporal bone, thyroid gland, and parotid glands [4]. The location of metastasis usually dictates the presenting symptoms.

Metastatic RCC will often behave similarly to that of the primary renal lesion in terms of morphology and histology. The risk of bleeding after fine needle aspiration biopsy of RCC in the kidney is well documented, with up to 90% of patients bearing evidence of perinephric bleeding on CT imaging and 5–7% developing clinically significant hemorrhage [5]. Therefore, clinical suspicion of RCC metastasis or other vascular lesions should prompt a biopsy in a controlled setting should hemorrhage ensue.

Histologically, differentiating among clear cell tumors with conventional light microscopy can be challenging. It can be especially difficult to distinguish between RCC metastasis and clear cell malignancies of salivary glands. Clear cell carcinomas of salivary gland origin are usually nests of clear cells divided by thin, fibrous connective septa and irregular vascular tissue. Immunohistochemical staining helps in this distinction, with RCC metastasis exhibiting focal cytokeratin positivity (versus minor salivary gland cancers showing diffuse positivity) and a strong reaction for vimentin [6].

Treatment of renal adenocarcinoma metastasis to the head and neck is directed mainly toward palliation [7]. Excision has been performed primarily to control pain and prevent bleeding and infection. Azam et al describe surgically debulking a rapidly growing metastatic tongue lesion in order to relieve pain and allow the patient to swallow. They then administered radiotherapy to the oral cavity at a dose of 60 Grays to treat any remaining microscopic disease [8]. Although RCC is traditionally known as a radioresistant tumor, radiotherapy can aid in local symptom control for perhaps a few months [2]. Little data exist regarding the use of systemic therapy in the setting of RCC metastasis to the oral cavity. Kyan and Kato report the surgical resection of a lingual mass followed by the administration of interferon-alpha and interleukin-II without recurrence of disease at 2 years [9]. Still, most patients die within 1 year after detection of head and neck metastasis; therefore, therapeutic decisions should maximize comfort and minimize morbidity considering the poor long-term prognosis at this stage of the disease.

Of interest in this patient is whether other measures taken at the time of the initial diagnosis and treatment period may have slowed disease progression or prolonged survival. Certainly, the finding of lymph node involvement portends a poor prognosis with 5- and 10-year survival rates similar to those with systemic metastasis (5–30% and 0–5%, respectively). An extended lymph node dissection has been suggested to benefit patients with early, isolated or microscopic involvement of the nodes. However, this remains controversial with a lack of randomized data to support any survival advantage [10].

Immunologic therapy following radical nephrectomy in the setting of metastatic disease may improve time to progression in properly selected patients. Prospective trials have demonstrated that, in patients with synchronous metastatic disease, cytoreductive nephrectomy and a systemic cytokine give a distinct survival advantage over those treated with immunotherapy alone (17.4 versus 11.7 months in patients with an Eastern Cooperative Oncology Group performance status of 0–1) [11]. Unfortunately, complete durable responses with systemic therapy are infrequent and the significant toxicity of immunologic agents may limit their use.

Finally, newer agents targeting the VEGF pathway such as bevacizumab and sorafenib may provide hope for patients with metastatic RCC. Early trials have shown a prolongation of progression-free survival with the use of these targeted molecular therapies in cytokine-refractory patients [12].

## Conclusion

RCC has been shown to metastasize to the head and neck region in rare instances. Therefore, the work-up of a new oral or neck lesion in light of a history of RCC should include metastatic RCC as part of the differential diagnosis. The physician needs to be prepared for the increased risk of bleeding involved in the biopsy of RCC metastasis. Should the biopsy specimen reveal clear cell carcinoma of the mouth, it is vital to perform immunohistochemical staining to differentiate between metastatic RCC and malignancies of salivary origin. If a diagnosis of metastatic RCC is established, additional therapeutic options, including immunotherapy, tyrosine kinase inhibitors, and participation in a clinical trial, should be discussed with the patient despite the poor overall prognosis.

## Abbreviations

CT: computed tomography; RCC: renal cell carcinoma; VEGF: vascular endothelial growth factor.

## Competing interests

The authors declare that they have no competing interests.

## Authors' contributions

TW collected and interpreted all of the urologic data pertinent to the case report and was the major contributing author of the final written report. NA collected and interpreted all of the otolaryngologic data pertinent to the case including the pathology specimens deemed Figures 1, 2, 3. GP performed the initial biopsy on the patient and critically revised the content and structure of the manuscript. All authors read and approved the final manuscript.

## Consent

Written informed consent was obtained from the patient for publication of this case report and accompanying images. A copy of the written consent is available for review by the Editor-in-Chief of this journal.
